# Stress distribution pattern in the distal radioulnar joint before and after ulnar shortening osteotomy in patients with ulnar impaction syndrome

**DOI:** 10.1038/s41598-021-97398-x

**Published:** 2021-09-09

**Authors:** Kazutoshi Hontani, Yuichiro Matsui, Daisuke Kawamura, Atsushi Urita, Daisuke Momma, Hiroki Hamano, Norimasa Iwasaki

**Affiliations:** 1 Department of Orthopaedic Surgery, Faculty of Medicine and Graduate School of Medicine, Hokkaido University, Kita 15, Nishi 7, Kita-ku, Sapporo, Hokkaido 060-8648 Japan; 2 Center for Sports Medicine, Hokkaido University Hospital, Sapporo, Hokkaido Japan

**Keywords:** Bone, Cartilage

## Abstract

Ulnar shortening osteotomy (USO) for ulnar impaction syndrome potentially leads to degenerative changes of the distal radioulnar joint (DRUJ). This study was performed to evaluate the effect of the sigmoid notch morphology on the stress distribution pattern of the DRUJ using computed tomography (CT) osteoabsorptiometry (CT-OAM). We reviewed the pre- and postoperative transverse CT images of 15 wrists that had undergone USO. The examined wrists were classified into two groups based on the sigmoid notch morphology: the linear-type notch (type L) and the curved-type notch (type C). We calculated and statistically compared the percentage of the high-density area (%HDA) in each divided region of the sigmoid notch. In type L, %HDA was significantly larger in the distal-dorsal region of the sigmoid notch before USO. Postoperatively, in type L, no specific regions showed a significantly different %HDA. In type C, %HDA was significantly larger in the distal-volar region of the sigmoid notch before USO. Postoperatively, %HDA of type C was significantly larger in the proximal-volar region. Our results suggest that in patients with ulnar impaction syndrome, morphological evaluation of the sigmoid notch can serve as a predictor of osteoarthritis in the DRUJ with or without USO.

## Introduction

Repeated excessive compression of the ulnar head against the triangular fibrocartilage complex (TFCC) and the ulnar carpal bones causes ulnar impaction syndrome, a complex, multifactorial disorder on the ulnar side of the wrist. Patients with ulnar impaction syndrome have ulnar-side wrist pain and a limited range of wrist motion. Ulnar impaction syndrome is diagnosed by clinical examination and radiographic assessment. Radiographic findings in patients with ulnar impaction syndrome frequently show positive ulnar variance^1^, but can also include neutral or negative ulnar variance with a thickened TFCC^2^.

Nonsurgical management such as wrist splinting is generally selected for patients with ulnar impaction syndrome. However, if conservative treatment fails, surgery to decompress the ulnocarpal space can be considered. Although ulnar shortening osteotomy (USO) is widely performed as a surgical treatment for ulnar impaction syndrome, several researchers have reported the occurrence of radiologic osteoarthritic changes in the distal radioulnar joint (DRUJ) after USO^3,4^. Previous studies reported that the radiologic development of osteoarthritic changes is associated with the amount of shortening and morphological variations in the DRUJ in the coronal plane^5–7^. However, there remains much to be understood regarding the effect of morphological variations in the DRUJ in the transverse plane on the occurrence of osteoarthritic changes in the DRUJ after USO.

In a cadaveric study, Nishiwaki et al.^8^ compared pressures at the DRUJ both before and after USO. They showed that the greater the ulnar shortening, the higher the peak pressure at the DRUJ. However, the mechanical effects of USO on the DRUJ in patients with ulnar impaction syndrome have not been fully elucidated. The DRUJ is a pivot joint that is composed of a sigmoid notch of the distal radius and ulnar head. DRUJ dorsopalmar translation occurs during rotation of the forearm as a consequence of the morphological features of the sigmoid notch because the notch is shallow and the curvature of the joint surface is approximately 50% greater than that of the ulnar head^9,10^. Because of these dynamic and morphologic features of the sigmoid notch, it is difficult to determine the changes in the stress distribution pattern at the sigmoid notch after USO in the living body using the conventional biomechanical method.

The subchondral bone density correlates with the stress distribution pattern that acts on a joint surface physiological loading conditions in vivo^11^. Previous studies demonstrated that subchondral bone mineral density measured using computed tomography (CT) osteoabsorptiometry (CT-OAM) can indicate long-term stress distribution patterns in joints^12^. We have evaluated stress distribution in various joints using this method^13–15^. Recently, the morphological differences in the sigmoid notch in the transverse plane were shown to affect the stress distribution pattern through the DRUJ in patients with carpal bone pathologies^6,7^. This method was considered a useful tool to the assess differences in the stress distribution patterns in the DRUJ both before and after USO in patients with ulnar impaction syndrome.

In this study, we hypothesize that the morphology of the sigmoid notch in the transverse plane affects the stress distribution pattern in the DRUJ with or without USO. To test this hypothesis, we employed the CT-OAM method to analyze the distribution of bone density in the sigmoid notch of the distal radius with or without surgery.

## Methods

### Patients

We retrospectively reviewed the pre- and postoperative transverse CT images of 15 wrists that had undergone USO for idiopathic ulnar impaction syndrome. The mean age of the patients at the time of surgery was 47.3 years (range, 27–63 years): nine right wrists and six left wrists were affected. The mean duration of evaluation was 21.9 months (range, 14–38 months) postoperatively. The exclusion criteria were as follows: pre-existing fracture of the radius or ulna, radiographic findings indicating osteoarthritis of the DRUJ, and history of any wrist surgery or systemic disease such as inflammatory arthritis.

### Surgical technique

All surgical procedures were performed under general anesthesia by three specialists in hand surgery (N.I., Y.M., D.K.). Details of the surgical procedure have been described previously^3,16^. After the arthroscopic procedures were complete, USO was performed by making two parallel transverse cuts to remove a segment of the bone, the length of which equaled the amount of positive ulnar variance measured on preoperative radiographs, taking into consideration the thickness of the saw blade. Fixation at the osteotomy site was accomplished using a six-hole small dynamic compression plate (LC-DCP; Synthes, West Chester, PA, USA). A volar short-arm splint was applied for 2 weeks after surgery, followed by active and passive motion according to the severity of pain. Full activity was resumed after bone healing, which took approximately 3 months.

### Classification of sigmoid notch morphology

**Figure 1:**
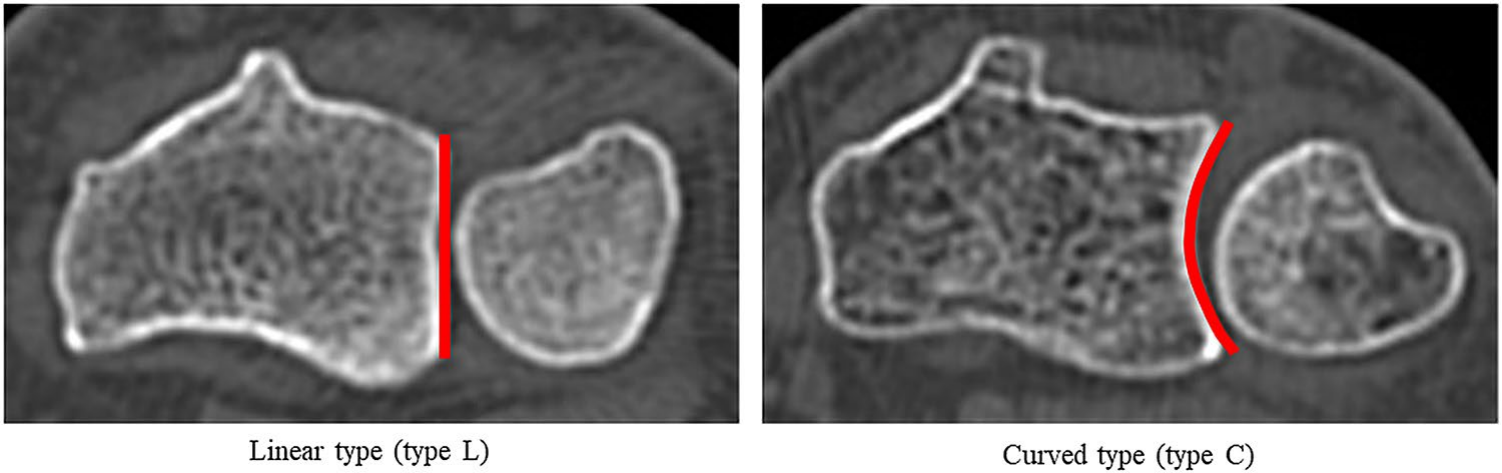
Typical computed tomographic images in the transverse plane of each type of sigmoid notch.

Using the transverse CT images of the DRUJ, we classified patients into two groups based on the type of the bone morphology of sigmoid notch at the level of Lister’s tubercle. In an anatomical study of 50 cadavers, Tolat et al.^17^ classified the bone morphology of the sigmoid notch into the following four types: flat-face notch (type FF), ski-slope notch (type SS), C-type notch (type C), and S-type notch (type S). In the present study, we attempted to categorize the bone morphology of the sigmoid notch in patients based on the classification of Tolat et al.^17^ However, in some cases, it was not clear whether the bone morphology was type FF or type SS, and no case was classified as type S. Therefore, we divided the morphology of the sigmoid notch into two types: (1) the linear-type notch (type L), including type FF and type SS, and (2) the curved-type notch (type C) (Fig. 1). The allocation of the sigmoid notch morphology was performed by two experienced orthopedic surgeons (K.H. and D.K.).

### Computed tomography osteoabsorptiometry

**Figure 2:**
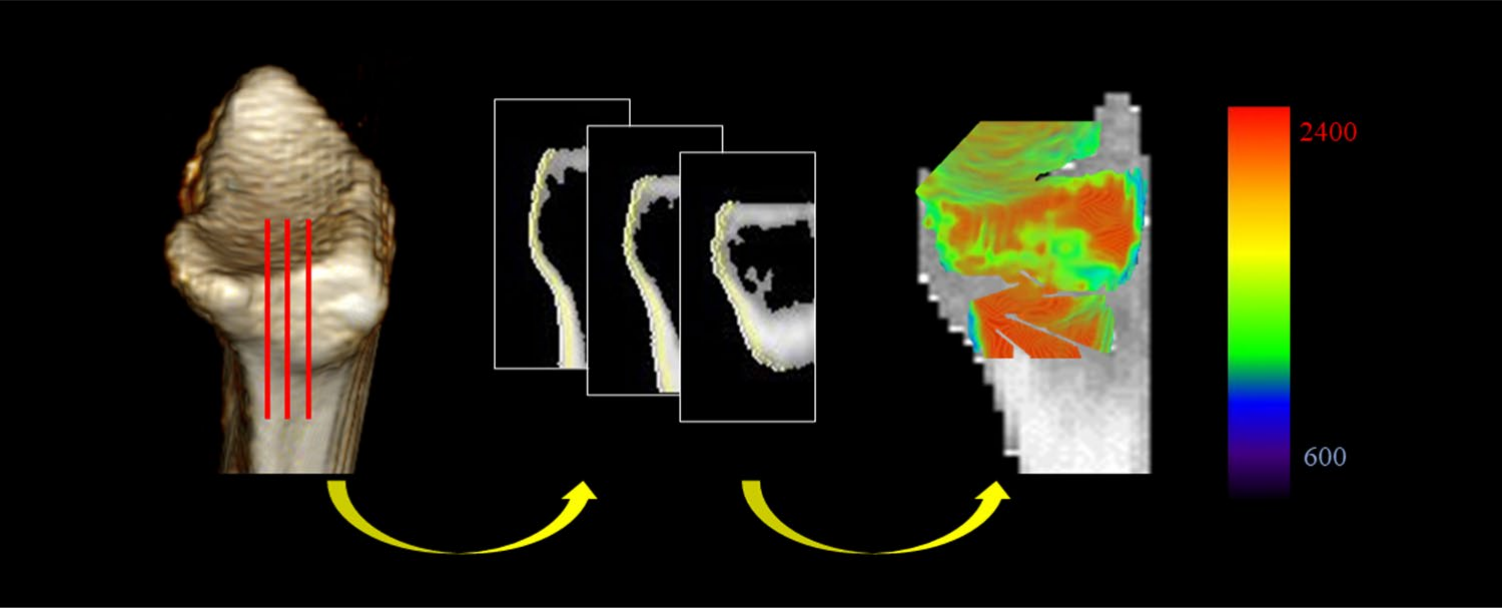
Identification of the subchondral bone regions of the sigmoid notch using customized software. In each coronal slice, X-ray absorption is measured at each coordinate point at 1-mm intervals. The surface-mapping image shows the distribution pattern in the sigmoid notch.

**Figure 3:**
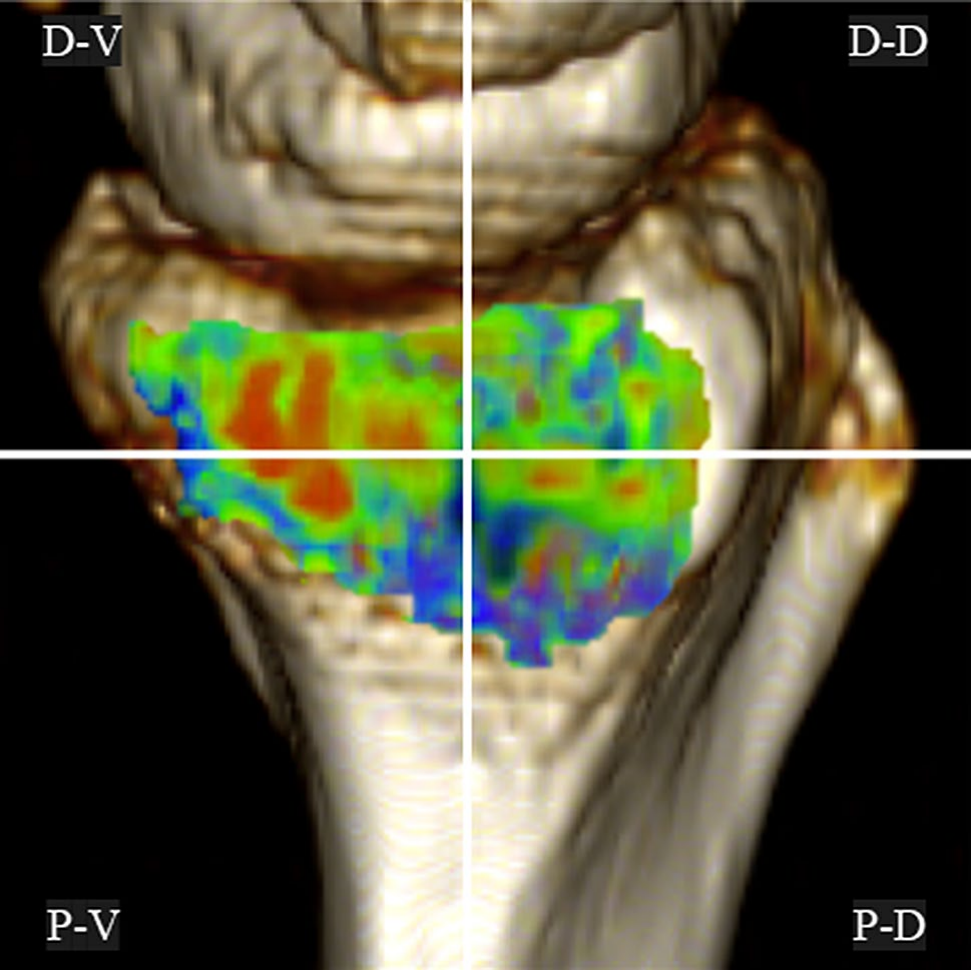
Four regions used for quantitative analysis of the bone density mapping data for the sigmoid notch. *D-V* Distal-volar, *D-D* Distal-dorsal, *P-V* Proximal-volar, *P-D* Proximal-dorsal.

**Figure 4:**
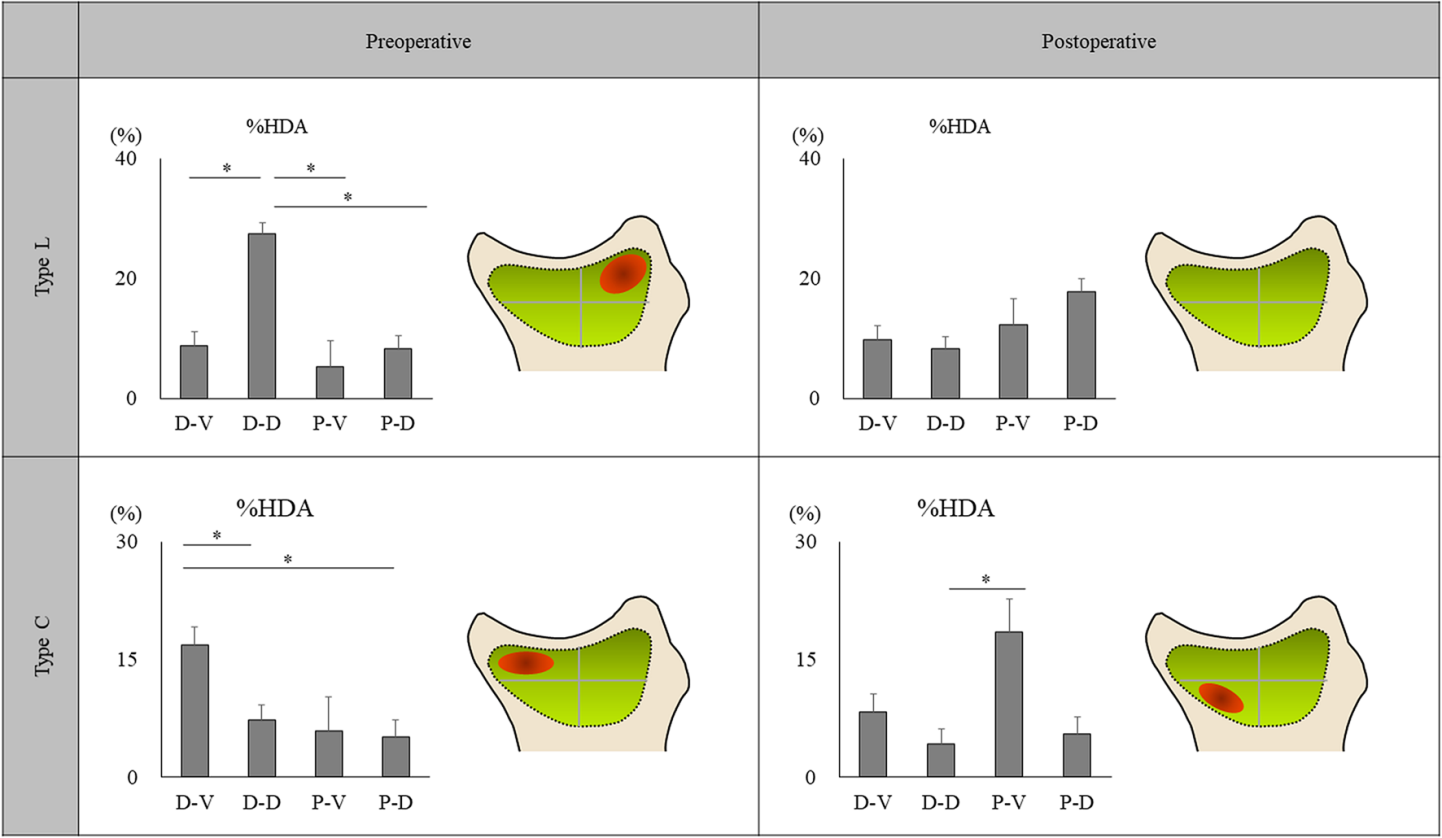
Distribution patterns of percentage high-density areas (%HDA) in type L and type C before and after surgery. The distribution of HDA in each region is shown schematically in the red circle in the figure. *D-V* Distal-volar, *D-D* Distal-dorsal, *P-V* Proximal-volar, *P-D* Proximal-dorsal. Statistical analysis was performed using the Kruskal–Wallis test (*p < 0.05).

Because it is technically difficult to evaluate the actual loading conditions in the DRUJ in vivo, we used CT-OAM to examine the distribution of the density of the subchondral bone on the articular surfaces of the sigmoid notch, which indicates the loading history. We used CT-OAM to evaluate the distribution of subchondral bone density, as previously described^13,14,18^. We constructed a three-dimensional bone model from the transverse image stack and then reconstructed sagittal views at 1-mm intervals from a multiplane reconstruction model. For further evaluation, we used a customized software program that we developed^13^. The region of interest was selected in the transverse images to include the entire subchondral bone layer of the proximal articular surfaces of the radiocarpal joint and ulnocarpal articulation in all slices. After establishing the region of interest, we automatically measured Hounsfield units (HU; defined as X-ray attenuation, in which water is 0 and compact bone is 1000) at each coordinate point within each 1-mm interval. Measurement and mapping were repeated in each slice. By stacking these data, a two-dimensional mapping image that projected the distribution of subchondral bone density in HU was obtained. We measured the subchondral bone densities of the sigmoid notch of the distal radius using the above-described techniques. The density range in HU from minimum to maximum in each wrist was divided into three equal intervals. The measured densities at each coordinate point were mapped with a three-grade color scale, with red indicating the highest bone density and deep blue indicating the lowest bone density (Fig. 2). We defined the high-density area (HDA) as the highest one-third of the entire range from minimum to maximum HU in each articulation. We measured the subchondral bone density within the articular surfaces of the sigmoid notch in the following four regions: distal-volar (D-V), distal-dorsal (D-D), proximal-volar (P-V), and proximal-dorsal (P-D) (Fig. 3). We then calculated the proportion of HDA on the joint surface (%HDA) for these regions and compared them statistically before and after surgery (Fig. 4). Intra-observer consistency in the measurement of subchondral bone density was assessed by calculating the coefficient of variation, which equals the standard deviation divided by the mean value, expressed as a percentage. One observer (K.H.) conducted five measurements of bone mineral density on a single CT image. We calculated the coefficient of variation of %HDA for the results of the five measurements.

### Statistical analysis

Statistical analysis was performed using the Kruskal–Wallis test. Differences were considered significant at *p* < 0.05. All statistical analyses were conducted using statistical software JMP Pro 10.0 (SAS Institute, Cary, NC, USA).

### Ethical approval

Ethics approval for this study was provided by the Institutional Review Board of Hokkaido University School of Medicine, and written informed consent was obtained from all patients prior to surgery. The methods were carried out in accordance with the principles of the Declaration of Helsinki.

## Results

Degenerative changes in the TFCC were identified during wrist arthroscopy in all patients. Arthroscopic examination showed degenerative changes (Class II according to Palmer’s classification) in the TFCC in all wrists. We intraoperatively confirmed the improvement of the stability after USO in all cases. The radiographic evaluation showed that no patients had postoperative osteoarthritic changes, including bony spur formation, within the follow-up period.

The coefficient of variation for intra-observer measurement of %HDA was 5.3%. Values less than 10% were considered acceptable for the purpose of performing comparisons^6,18^. All %HDA measurements were conducted by a single observer. There were 10 type L and 5 type C notches in this study. Table 1 shows the %HDA, and Fig. 4 presents the distribution patterns of %HDA in type L and type C both before and after surgery.

**Table 1:** Percentage of the high-density area with SDs of each region in the sigmoid notch according to the type of bony morphology.

Region	%HDA (SD)			
	Type L		Type C	
	Preoperative	Postoperative	Preoperative	Postoperative
D-V	8.7 (2.7)	9.8 (2.5)	16.8 (2.3)	8.3 (3.4)
D-D	27.4 (5.4)	8.3 (3.1)	7.2 (20.0)	4.2 (2.2)
P-V	5.3 (1.5)	12.3 (4.2)	5.9 (4.2)	18.5 (6.0)
P-D	8.2 (2.7)	17.7 (5.5)	5.1 (2.2)	5.5 (2.4)

*D-V* Distal-volar, *D-D* Distal-dorsal, *P-V* Proximal-volar, *P-D* Proximal-dorsal, *%HDL* Percentage high-density area, *SD* Standard deviation.

In type L, %HDA was significantly larger in the D-D region of the sigmoid notch than in the other regions before surgery, whereas no specific region had significantly different %HDA after surgery (Table 2). In type C, %HDA was significantly larger in the D-V region of the sigmoid notch than in the dorsal regions (D-D and P-D) before surgery. %HDA was significantly larger in type C after surgery in the P-V region than in the D-D region and tended to be larger than in the other two regions (D-V and P-D).

**Table 2:** Comparative analysis of each region of the sigmoid notch before and after surgery.

Standard region	Control region	*p* value			
		Type L		Type C	
		Preoperative	Postoperative	Preoperative	Postoperative
D-V	D-D	0.005*	0.571	0.022*	0.346
D-V	P-V	0.471	0.940	0.143	0.144
D-V	P-D	0.910	0.385	0.022*	0.753
D-D	P-V	< 0.001*	0.545	0.295	0.037*
D-D	P-D	0.005*	0.241	0.403	0.753
P-V	P-D	0.520	0.678	0.675	0.060

Kruskal–Wallis test (*p < 0.05).

*D-V* Distal-volar, *D-D* Distal-dorsal, *P-V* Proximal-volar, *P-D* Proximal-dorsal.

## Discussion

Ulnar impaction syndrome causes osteoarthritis of the DRUJ as it progresses. Osteoarthritis of the DRUJ has also been reported as a complication after USO^16,19^. The bone morphology of the DRUJ and alterations of the stress distribution pattern within it are two factors that result in osteoarthritis^5,20,21^. We hypothesized that the morphology of the sigmoid notch in the transverse plane affects the stress distribution pattern in the DRUJ. In the present study, we used CT-OAM to examine the stress distribution pattern in the DRUJ in patients with ulnar impaction syndrome both before and after surgery, with emphasis on the morphology of the sigmoid notch.

**Figure 5:**
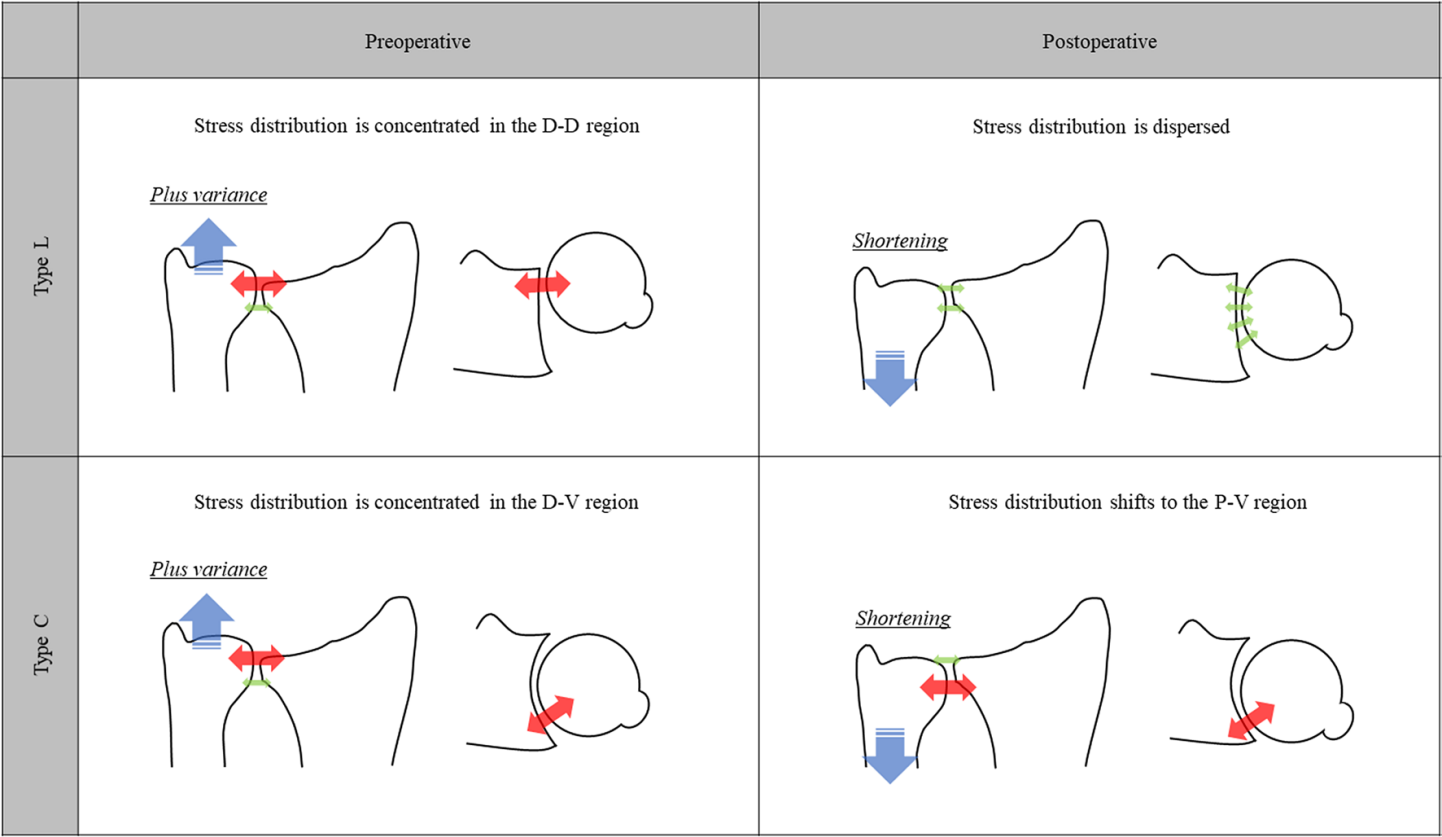
Mechanism of stress distribution in each bone morphological type of the sigmoid notch before and after surgery. *D-V* Distal-volar, *D-D* Distal-dorsal, *P-V* Proximal-volar.

Tolat et al.^17^ reported that, of the various appearances of the sigmoid notch in the transverse plane, type FF could be considered the least congruous and potentially more unstable. When the joint surface of the sigmoid notch has linear morphology, there is an inclination with respect to the volar cortex of the radius on the transverse plane^6^. This morphological congruity of the DRUJ may result in relatively easy mobility of the ulnar head toward the dorsal side of the radius, resulting in a concentration of stress in a narrow area on the dorsal side of the radius (Fig. 5). Nakamura et al.^22^ reported that when the hammock structure of the TFCC is pulled down by USO, the TFCC becomes tensioned, thereby stabilizing the DRUJ. In a study using cadavers, Nishiwaki et al.^8^ reported that the contact area of the DRUJ expanded when the ulna was shortened. Considering these findings of the above-mentioned studies, the current study indicated that the stress distribution in the dorsal side of the sigmoid notch in type L is likely dispersed postoperatively (Fig. 5). However, Tolat et al.^23^ examined the wrist morphology of patients with carpal bone pathologies and reported that the type C sigmoid notch has a regular curved shape. In our study, similar to that of Tolat et al.^23^, the bone morphology of the type C sigmoid notch had an arc-like shape on the transverse plane. Therefore, the ulnar head contacted the bone tissue overhanging the palm ulnar side of the sigmoid notch, possibly resulting in stress concentration in this area (Fig. 5). Deshmukh et al.^4^ reported that the congruity of the DRUJ after USO was affected by the morphology of the sigmoid notch and ulnar head. Based on this finding, we speculated that when the ulna was shortened, the ulnar head would move toward the proximal palmar side, causing stress concentration in the P-V region of the sigmoid notch (Fig. 5). Thus, based on these results, for patients with ulnar impaction syndrome, differences in the morphology of the sigmoid notch on the transverse plane affect the stress distribution pattern in the DRUJ.

In a study using cadavers, Nishiwaki et al.^21^ reported that USO resulted in increased pressure on the DRUJ. However, stress distribution pattern changes in the DRUJ after USO in vivo have not been fully elucidated. According to the already known biomechanical features of the DRUJ, forearm pronation and supination cause not only compressive force but also shearing force on the sigmoid notch^9,10^. Therefore, analyzing the stress distribution pattern in the DRUJ in vivo using conventional biomechanical methods is difficult. CT-OAM is a technique to predict the stress distribution pattern in a joint in vivo by measuring the mineral density of the subchondral bone on the basis of CT data^12^. Therefore, we attempted to use CT-OAM to determine the mechanical effects of USO on the DRUJ in patients with ulnar impaction syndrome. We acquired CT images of the wrist before and after surgery in patients with ulnar impaction syndrome. We assumed that the external forces on the wrist during daily activities were reduced by immobilization or protection of the operated wrist during the perioperative period. However, a long postoperative period would lead to difficulties in evaluating the mechanical effects of the surgery. To eliminate these factors, we analyzed the CT image data obtained from all patients after at least 14 months and before 38 months postoperatively.

Stress concentration on the articular surface has been suggested to induce osteoarthritis^24^. Giunta et al.^25^ analyzed the stress distribution pattern in the DRUJ in 22 healthy wrist joints using CT-OAM. They reported that stress was concentrated distally on the dorsal side of the sigmoid notch in 45% of the cases and on the palmar side in 36% of the cases. They stated that the physiological incongruence of the articulating bony curvature and the volar dorsal translational movement led to very limited contact areas of the sigmoid notch. In this study, the preoperative results indicated that when USO is not performed on patients with ulnar impaction syndrome, osteoarthritis may occur in the future in the D-D region in type L and in the D-V region in type C. However, osteoarthritis of the DRUJ has been reported as a long-term postoperative complication of USO. Iwasaki et al.^19^ reported osteoarthritis of the DRUJ in 34% of cases at 26 months after USO on average. However, the mechanism of occurrence of osteoarthritis in the DRUJ after USO remains unclear. The present study results suggest that, in type L cases, the risk for osteoarthritis on the dorsal side of the sigmoid notch was reduced by USO, whereas in type C cases, the risk for osteoarthritis on the proximal palmar side of the sigmoid notch might be increased. These results suggest that in patients with ulnar impaction syndrome, stress distribution in the DRUJ is affected by the morphology of the sigmoid notch on the transverse plane and that the morphology serves as a predictor of osteoarthritis in the DRUJ after USO.

The present study has several limitations. First, whether there exists a correlation between the morphology of the sigmoid notch on the transverse plane and the coronal plane remains unclear. Second, the CT-OAM used in the present study is not capable of measuring the absolute stress, although it is an excellent evaluation tool that enables the analysis of the relative stress distribution in vivo. Third, we could not accurately evaluate whether the degree of damage of the TFCC, the degree of stability of the DRUJ before and after surgery, and the amount of ulnar shortening influenced the patterns of the stress distribution in the DRUJ in our study. Fourth, because none of the patients developed radiologically confirmed osteoarthritis of the DRUJ during the study period, we could not evaluate whether changes in the stress distribution really cause degenerative changes. Further studies including a larger number of patients with long-term radiologic follow-up are necessary to demonstrate the association between the occurrence of postoperative osteoarthritis and the morphology of the transverse and coronal plane of the sigmoid notch.

Using CT-OAM, we evaluated the patterns of stress distribution in the DRUJ both before and after USO for ulnar impaction syndrome. In the linear-shaped sigmoid notch, the stress distribution within the DRUJ before surgery was concentrated in the distal-dorsal region, whereas the stress was distributed uniformly in the postoperative period. In the curved-shaped sigmoid notch, the stress was concentrated in the distal-volar region as compared with the dorsal regions before USO, whereas, after surgery, the stress concentration tended to shift proximally.

These results suggest that when USO is performed, the risk for the degenerative change on the dorsal side of the linear-shaped sigmoid notch is reduced and the risk for degenerative change on the proximal palmar side of the curved-shaped sigmoid notch is increased. Thus, morphological evaluation of the sigmoid notch on the transverse plane may predict where the degenerative change is likely to occur in the DRUJ in patients with ulnar impaction syndrome with or without surgery.
